# Anxiety, Insomnia, and Napping Predict Poorer Sleep Quality in an Autistic Adult Population

**DOI:** 10.3390/ijerph18189883

**Published:** 2021-09-19

**Authors:** Emma C. Sullivan, Elizabeth J. Halstead, Jason G. Ellis, Dagmara Dimitriou

**Affiliations:** 1 Sleep Education and Research Laboratory, Department of Psychology and Human Development, Institute of Education, University College London, London WC1H 0AA, UK; emma.sullivan@ucl.ac.uk; 2 Northumbria Sleep Research Laboratory, Department of Psychology, Northumbria University, Newcastle upon Tyne NE1 8ST, UK; jason.ellis@northumbria.ac.uk

**Keywords:** sleep quality, daytime sleepiness, autistic adults, demographic factors, lifestyle factors

## Abstract

Autistic adults have a high prevalence of sleep problems and psychiatric conditions. In the general population sleep problems have been associated with a range of demographic and lifestyle factors. Whether the same factors contribute to different types of disturbed sleep experienced by autistic adults is unknown and served as the main aim of this study. An online survey was conducted with 493 autistic adults. Demographic information (e.g., age, gender), about lifestyle (e.g., napping), and information about comorbid conditions was collected. The Pittsburgh Sleep Quality Index (PSQI) was used to assess sleep quality and the Epworth Sleepiness Scale (ESS) was used to assess daytime somnolence. Stepwise multiple regression analyses were used to examine predictors of each subscale score on the PSQI, as well as PSQI and ESS total scores. Results indicated that individuals who reported having a diagnosis of anxiety and insomnia were more likely to have poorer sleep quality outcomes overall. Furthermore, individuals who reported habitually napping had higher daytime dysfunction, increased sleep disturbances, and increased daytime sleepiness. These results provide novel insights into the demographic and lifestyle factors that influence sleep quality and daytime somnolence in autistic adults and can be used for targeted sleep interventions.

## 1. Introduction

In comparison to the general population, autistic adults have been found to have a higher prevalence of sleep problems, including longer sleep latencies, a higher number, and duration, of night waking occurrences, shorter night sleep durations, disrupted circadian rhythms, and greater levels of daytime sleepiness [1,2,3]. Furthermore, they are at a higher risk of developing psychiatric conditions such as depression, anxiety, and bipolar disorder, compared to the general population [4,5]. As in the general population, the reasons for sleep problems are likely due to multifaceted interactions between environmental and biopsychological factors. However, whether any of these factors map onto the same socio-demographic, economic, and lifestyle factors implicated in the general population remains unknown.

In the general population, poor sleep has been associated with a range of factors such as age, gender, and socioeconomic status (SES) [6,7,8]. For example, ageing has been associated with changes in sleep physiology and diminished sleep quality [9,10,11,12]. In a meta-analysis conducted by Ohayon and colleagues [10], it was reported that total sleep time, sleep efficiency, percentage of time spent in different sleep stages, and REM latency all decreased with age, whereas sleep latency, percentage of time spent in stage 1 and stage 2 sleep, and wake after sleep onset all increased with age. Moreover, increased levels of daytime sleepiness have also been associated with increased age [13,14]. In addition, women tend to report more sleep problems, insufficient sleep, and have a higher risk of developing insomnia than men [6,15,16]. Arber and colleagues [6] found an association between sleep disturbances and household income, education qualifications, unemployment, and housing status. Higher odds of reported sleep difficulties among unemployed and low-educated adults were also observed. Furthermore, the authors reported that SES inequalities may play a major part in women reporting more frequent sleep problems than men [6]. This link between sleep and SES in the general population is consistent with previous research [14,15,17,18,19,20,21,22].

Lifestyle factors might also be important contributors to sleep quality. Factors that have been associated with poor sleep in the general population include smoking, alcohol, exercise, diet, and napping. Frequent smoking and alcohol consumption have been found to have a negative impact on sleep quality [23,24,25,26,27,28,29]. In addition, smoking has also been associated with insomnia and shorter sleep durations [30].

Conversely, engaging in regular physical exercise has been found to have a positive influence on sleep quality [31,32,33,34], and better sleep quality has been found to be associated with good diet [35,36,37]. The effects of habitual napping on sleep quality are mixed, with some finding that napping improves nocturnal sleep quality [38] and others reporting a negative effect on nocturnal sleep quality [39] or finding no effect [40]. Taken together, these findings implicate an important role of sociodemographic, socioeconomic, and lifestyle factors in contributing to sleep quality outcomes in the general population.

In autistic adults, far less is known about the demographic and lifestyle factors relating to sleep quality and daytime sleepiness. Limited research has implicated SES as a contributor of poor sleep quality in autistic adults. For example, Baker et al. [41] found autistic adults with diagnosed sleep disorders were more likely to be unemployed compared to autistic adults with no diagnosed sleep disorders. It was suggested that unemployment may be one factor contributing to poor sleep in this population, possibly through the dysregulation of sleep schedules. However, daytime sleepiness was not significantly associated with unemployment in this study. As the above study focused largely on sleep quality, the demographic and lifestyle factors contributing to daytime dysfunction and daytime sleepiness in an autistic adult population require additional investigation. In sum, despite some evidence, it remains largely unknown whether factors contributing to sleep quality and daytime sleepiness in autistic adults are equivalent with those found to be important in the general population.

With this in mind, investigating whether these aforementioned factors predict sleep quality and daytime sleepiness in the autistic population is an important research endeavour. Given that poor sleep quality and mental health problems are more prevalent in this population, it is possible that autistic adults may resort to coping mechanisms such as alcohol and smoking more frequently when compared to the general population. Furthermore, demographic and lifestyle factors may contribute to sleep quality and daytime sleepiness differently in this population. For example, autistic adults’ preferences and frequency of leisure participation has been shown to differ from that of the general population. Compared to a neurotypical population, autistic adults reported less satisfaction with leisure activities and were less likely to attend social events [42]. In addition, being younger and reporting less symptoms of depression was associated with higher leisure satisfaction in autistic adults. These findings suggest that factors such as physical exercise may not provide the same benefits as shown in the general population, with ageing and higher levels of depression contributing to this lack of satisfaction. As a result, identifying the demographic and lifestyle factors that may impact on sleep quality and daytime sleepiness in autistic adults can help in the development of tailored sleep interventions that improve sleep quality and, subsequently, quality of life. Given the limited research on sleep quality predictors, the current study aimed to determine which demographic and lifestyle factors were associated with sleep quality and daytime sleepiness in an autistic adult population.

## 2. Materials and Methods

### 2.1. Participants

Participants were recruited for an online survey, between November 2019 to February 2020, via social media and autism support groups. All adults that identified as an autistic adult were included in our sample to allow access to a more diverse population and reduce barriers related to accessing an autism diagnoses in adulthood [43,44]. Of the 837 UK participants that responded to the online Qualtrics^XM^ survey, 58.90% fully completed the survey (*n* = 493 final sample).

All participants identified as an autistic adult (408 were diagnosed by a healthcare professional, 85 were self-diagnosed). Participants who reported a diagnosis by a healthcare professional (HCP) were asked for their age at diagnosis, the type of professional who diagnosed them (e.g., psychiatrist), and the date of their diagnosis (month, year). Autistic adults who completed the survey were predominantly female (56.59% females, 24.34% male, 16.84% non-binary, 2.23% prefer not to say). Participants were aged between 18–73 years old (*M* = 36.12, *SD* = 11.40). Additional demographic information is included in Table 1. See Supplementary Materials Tables S1–S3 for further demographic and clinical information, including co-morbidities.

### 2.2. Materials and Design

#### 2.2.1. Demographic Questionnaire

The demographic survey included reports of current autism diagnosis (self-report or diagnosed by an HCP), age, gender, ethnicity, education, employment status, number of dependents, and postcode. We also included questions regarding lifestyle habits, such as smoking, alcohol use, exercise and habitual napping. Questions regarding lifestyle habits were rated on a Likert scale. For example, exercise frequency was rated from 1–6 (1—everyday; 2—≥5x week; 3—≥3x a week; 4—≥1x a week; 5—<1x a week; 6—less than once a month). See Supplementary Materials Table S1 for characterisation of lifestyle habits.

Several indicators were combined to create an index of socioeconomic status (SES; [45]). The first indicator was annual household income, which was scored from 1–8, with higher values indicating a lower average annual household income (calculated from the 2021 census data; [46]). Education level was scored 1 (college education or below) or 0 (university education or above). Employment status was scored 1 (no employment) or 0 (employed, full or part time or self-employed). Total SES was calculated by summing the scores of these four indicators (range 1–10). The highest score of 10 indicated earning less than £20,000 a year, having educational qualifications below degree level, and being unemployed. We did not include an indicator of neighbourhood deprivation in our index of SES given that 70.00% of our total sample did not provide postcode information.

#### 2.2.2. Sleep Quality

Sleep quality was measured using the Pittsburgh Sleep Quality Index (PSQI); a 19-item self-report questionnaire measuring sleep quality over the previous month [47]. Scoring on the items ranges from 0 (not during the past month) to 3 (three or more times during the past month). Items included in the PSQI measure sleep disturbances across seven dimensions: (1) subjective sleep quality, (2) sleep latency, (3) sleep duration, (4) habitual sleep efficiency, (5) sleep disturbances, (6) use of sleep medication, and (7) daytime dysfunction. These subscales are summed to produce a global PSQI score, which ranges from 0–21. Higher global scores on the PSQI indicate worse sleep quality, with a cut off score of >5 indicating poor sleep quality. In the current study, the PSQI demonstrated adequate internal consistency for the seven dimensions (Cronbach’s α = 0.68).

#### 2.2.3. Daytime Sleepiness

Daytime sleepiness was assessed using the Epworth Sleepiness Scale (ESS). The ESS is an 8-item, self-administered questionnaire designed to evaluate overall daytime sleepiness [48]. Participants rate the chances of dozing or falling asleep while engaged in eight different activities from 0 (would never doze) to 3 (high chance of dozing). Total ESS scores range from 0 to 24. Higher scores indicate higher levels of daytime sleepiness. A score above 10 is considered to be pathological sleepiness, as previously validated [48]. The ESS has been used previously to investigate sleep disturbances in young autistic adults [49]. In the current study, the ESS demonstrated good internal consistency (Cronbach’s α = 0.83).

### 2.3. Procedure

Participants were invited to complete an online survey through a multi-point recruitment method, which included emailing online links and distributing advertisements and information sheets to UK charities relevant to autistic adults and autistic adult support groups. Online recruitment via social media (Twitter, Facebook, and Instagram) was also ongoing throughout the recruitment period [November 2019–February 2020]. Prior to consent, participants were presented with a brief introduction to the survey and given the opportunity to read the information sheet. Informed consent was then obtained from all participants prior to completing the survey.

### 2.4. Analysis

Based on the aforementioned literature, a number of demographics and lifestyle variables were included in in our statistical analysis and are reported below. Continuous variables of interest included age and SES ranking. All categorical variables of interest were recoded into binary variables. Binary variables included gender (male vs. not male, female vs. not female, binary vs. non-binary), ASD diagnosis (diagnosed by a HCP vs. self-diagnosed), exercise presence (yes/no), exercise frequency (high exercise frequency: ≥3 times a week vs. low exercise frequency: <3 times a week), alcohol presence (yes/no), alcohol frequency (high alcohol frequency: ≥1 unit a day vs. low alcohol frequency <1 unit a day), and habitual napping presence (yes/no). Binary variables such as exercise frequency were estimates of averages in a typical week. Binary predictor values with <10% minority in either group (i.e., smoking presence and napping duration) were not included in our final analyses. Given that insomnia is a common source of distress in this population, we also included diagnosis of insomnia (yes/no) as a covariate in our statistical analysis. In addition, to control for the influence of comorbid mental health conditions, we included any diagnosed conditions (yes/no) reported in >50% of the population as covariates in our main analyses, these included depression and anxiety. An a priori power analysis was conducted in G*Power 3.1.9.7 software [50] to determine the required effect sizes for each of the outcome variables in the current study. Given the known sample sizes in each of our outcome variables, with 80% power and α = 0.05, we were able to detect a minimum effect size of Cohen’s F^2^ = 0.07. This demonstrated that we were sufficiently powered to detect minimal effect sizes with our sample size.

All subscales and total scores were not normally distributed (SW *p* < 0.05), therefore non-parametric tests were conducted. Spearman correlations were used to investigate whether there were any significant associations between continuous variables of interest (SES, age), PSQI subscale and global scores, and ESS total score. In the case of binary variables of interest, Mann–Whitney U tests were conducted to investigate whether there were any significant differences between two independent groups (e.g., males vs females; exercise presence vs no exercise) on the PSQI subscale and global scores and ESS total score. In order to examine whether demographic and lifestyle variables were significant predictors of sleep quality, we next conducted separate multiple linear regressions for each outcome measure (PSQI subscale and global scores, ESS total score) using our variables of interest. Given the exploratory nature of this research, we used the stepwise method as our entry method in each of our regression analyses. The significance threshold for all analyses was set at 0.05.

### 2.5. Missing Data

Missing data on some of the PSQI subscales and PSQI total outcome scores was a result of either participant error or nonresponse on one or more of the individual items used to calculate these scores. For example, several participants reported getting more than 24 h of sleep in one night, making the sleep duration subscale incomputable. In these cases, a missing value replaced the response. The sample sizes for each outcome measure are reported in Table 2. In addition, some predictor responses (e.g., exercise frequency) were only based on a subset of the sample (i.e., those who reported exercising), limiting the number of participants with complete predictor data in the regression analyses. Final sample sizes for each regression analysis are provided in the Supplementary Materials Table S4.

## 3. Results

### 3.1. Descriptive Data

Mean scores for each outcome measure are reported in Table 2. Associations between all predictor variables to be included in our regression models and sleep quality outcome variables were examined first. Increased age was associated with a lower sleep latency score (r_s_ = −0.09, *p* = 0.043), a higher sleep duration score (r_s_ = 0.19, *p* < 0.001), and a higher sleep disturbance score (r_s_ = 0.12, *p* = 0.009). Higher SES scores were associated with a higher sleep latency score (r_s_ = 0.20, *p* < 0.001), a higher sleep disturbance score (r_s_ = 0.13, *p* = 0.006), a higher daytime dysfunction score (r_s_ = 0.16, *p* = 0.001), and a higher overall sleep quality score (r_s_ = 0.12, *p* = 0.019). Males (vs. female and nonbinary) scored significantly lower on the sleep disturbance subscale (U = 19,216.00, *p* = 0.009). Non-binary participants (vs. female and male) scored significantly lower on the sleep duration subscale (U = 13,998.00, *p* = 0.036) and females (vs. nonbinary and males) scored significantly higher on the sleep duration subscale (U = 25,334.00, *p* = 0.024). For the sleep medication subscale of the PSQI, males scored significantly lower (U = 19,229.00, *p* = 0.007) whereas females scored significantly higher (U = 27,119.50, *p* = 0.042) compared to the other two groups. Participants who were diagnosed as autistic by an HCP scored significantly higher on the subjective sleep quality subscale compared to those who were self-diagnosed (U = 15,180.00, *p* = 0.048).

Participants who exercised (vs. no exercise) scored significantly lower on the sleep latency subscale and the daytime dysfunction subscale (U = 25,341.00, *p* = 0.032; U = 26,606.50, *p* = 0.028, respectively). Participants who reported exercising frequently (≥3x week) had a significantly lower ESS total score compared to those who reported exercising less frequently (<3x week; U = 7385.50, *p* = 0.047). There were no significant differences between participants who reported consuming alcohol vs. those who reported not consuming alcohol and participants who reported frequently consuming alcohol (≥1 unit a day) vs. those who reported less frequently consuming alcohol (<1 unit a day) on any of the sleep quality outcome measures (*p* > 0.05).

Participants who reported to habitually nap (vs. not habitually nap) scored significantly higher on the subjective sleep quality subscale (U = 19,896.50, *p* = 0.028), the sleep duration subscale (U = 17,950.50, *p* = 0.006), the sleep disturbance subscale (U = 16,616.00, *p *< 0.001), and the daytime dysfunction subscale (U = 15,692.00, *p* < 0.001). PSQI total score (U = 16,334.00, *p* = 0.003) and ESS total score (U = 11,438.50, *p* < 0.001) were also higher in those that reported to habitually nap, compared to those who did not report habitually napping. See Table 3 for reported medians across groups and Table 4 for an overview of significant associations between demographic and lifestyle variables and sleep quality and daytime sleepiness outcomes.

### 3.2. Multiple Regression Analysis: Examining Predictors of Sleep Quality

Multiple regression analyses were conducted using the stepwise procedure to investigate whether the demographic and lifestyle variables of interest predicted sleep quality, as indexed by PSQI subscales and global score and ESS total score. All significant predictor coefficients for each of the regression models are displayed in Table 5.

Subjective Sleep Quality: The results of the regression indicated that the final predictors explained 8.5% of the variance. R² = 0.085, (adjusted R² = 0.078, F(2,276) = 12.81, *p* < 0.001, Cohen’s ƒ^2^ = 0.09). The analysis indicated that both insomnia diagnosis (β = 0.19, *p* = 0.001) and anxiety diagnosis (β = 0.19, *p* = 0.002) significantly predicted subjective sleep quality score.

Sleep Latency: The results of the regression indicated that the final predictors explained 8.4% of the variance, R² = 0.084 (adjusted R² = 0.074, F(3,267) = 8.19, *p* < 0.001, Cohen’s ƒ^2^ = 0.09). The analysis indicated that anxiety diagnosis (β = 0.16, *p* = 0.010) and insomnia diagnosis (β = 0.17, *p* = 0.004) and age (β = −0.14, *p* = 0.019) were both significant predictors of sleep latency score.

Sleep Duration: The results of the regression showed that the final predictors explained 4.2% of the variance, R² = 0.042 (adjusted R² = 0.035, F(2,272) = 5.91, *p* = 0.003, Cohen’s ƒ^2^ = 0.04). The analysis revealed that anxiety diagnosis (β = 0.16, *p* = 0.009) and age (β = 0.15, *p* = 0.015) significantly predicted sleep duration score.

Sleep Efficiency: The results of the regression showed that the final predictors explained 4.4% of the variance, R² = 0.044 (adjusted R² = 0.037, F(2,264) = 6.13, *p* = 0.003, Cohen’s ƒ^2^ = 0.05). The analysis revealed that anxiety diagnosis (β = 0.15, *p* = 0.016) and insomnia diagnosis (β = 0.12, *p* = 0.047) significantly predicted sleep efficiency score.

Sleep Disturbance: The results of the regression demonstrated that the final predictors explained 7.8% of the variance, R² = 0.078 (adjusted R² = 0.068, F(3,275) = 7.74, *p* < 0.001, Cohen’s ƒ^2^ = 0.08). Insomnia diagnosis (β = 0.18, *p* = 0.003), napping presence (β = 0.16, *p* = 0.007), and anxiety diagnosis (β = 0.12, *p* = 0.042) all significantly predicted sleep disturbance score.

Sleep Medication: The results of the regression demonstrated that the final predictors explained 8.9% of the variance, R² = 0.089 (adjusted R² = 0.086, F(1,277) = 27.14, *p* < 0.001, Cohen’s ƒ^2^ = 0.10). Insomnia diagnosis (β = 0.30, *p* < 0.001) was the only significant predictor of sleep medication score.

Daytime Dysfunction: The results of the regression indicated that the final predictors explained 12.6% of the variance, R² = 0.126 (adjusted R² = 0.116, F(3,275) = 13.19, *p* < 0.001, Cohen’s ƒ^2^ = 0.14). Depression diagnosis (β = 0.15, *p* = 0.026), napping presence (β = 0.19, *p* = 0.001), and anxiety diagnosis (β = 0.16, *p* = 0.022) were all found to significantly predict daytime dysfunction score.

Overall Sleep Quality: The results of the regression demonstrated that the predictors explained 14.3% of the variance, R² = 0.143 (adjusted R² = 0.137, F(2,262) = 21.89, *p* < 0.001, Cohen’s ƒ^2^ = 0.17). The analysis indicated that both insomnia diagnosis (β = 0.28, *p* < 0.001) and anxiety diagnosis (β = 0.21, *p* < 0.001) significantly predicted overall sleep quality score.

Daytime Sleepiness: The results of the regression indicated that the final predictors explained 13.4% of the variance, R² = 0.134 (adjusted R² = 0.131, F(1,277) = 42.90, *p* < 0.001, Cohen’s ƒ^2^ = 0.15). The analysis indicated that napping presence (β = 0.37, *p* < 0.001) was the only significant predictor of daytime sleepiness. We also conducted multinomial logistic regression analysis to test if daytime sleepiness was associated with napping duration (see Supplementary Materials Table S1 for groupings). The results of the regression analysis indicated that daytime sleepiness did not significantly predict napping duration, Nagelkerke *R*^2^ = 0.01, χ^2^(5) = 1.52, *p* = 0.910.

## 4. Discussion

Given the higher prevalence of co-morbid sleep and mental health problems in autistic adults, the aim of the present study was to investigate factors that could impact on sleep quality and daytime sleepiness in this population. The findings suggest that anxiety and insomnia diagnoses are both associated with poorer sleep quality in this population. In addition, habitual napping is associated with higher daytime sleepiness. Different lifestyle factors were found to be predictive of different types of poor sleep quality. For example, a diagnosis of anxiety and/or insomnia related to a longer sleep latency and lower sleep efficiency, whereas habitual napping was related to higher daytime sleepiness.

Unsurprisingly, we found that insomnia diagnosis was a significant predictor of poor sleep quality, with the exception of sleep duration, daytime dysfunction, and daytime sleepiness. Therefore, participants who reported a diagnosis of insomnia (vs. participants who did not report a diagnosis of insomnia) had lower subjective sleep quality, longer sleep latencies, lower sleep efficiency, higher sleep disturbances, and lower overall sleep quality. This result was expected given that insomnia is a common source of distress in the autistic adult population, with a higher prevalence compared to the general population [51].

In addition, an anxiety diagnosis was a significant predictor of all the sleep quality outcome measures, with the exception of sleep medication and daytime sleepiness. Therefore, participants who reported a diagnosis of anxiety (vs. participants who did not report a diagnosis of anxiety) had lower subjective sleep quality, longer sleep latencies, shorter sleep duration, lower sleep efficiency, higher sleep disturbances, and higher daytime dysfunction. They also had lower overall sleep quality. This is concordant with findings that anxiety disorder is frequently associated with poor sleep [52,53,54]. Given that autistic adults have a higher prevalence of anxiety than the general population [4,5], it is plausible that anxiety may contribute to poorer sleep quality outcomes above and beyond any demographic or lifestyle factors associated with poorer sleep quality in the general population [1]. Moreover, a depression diagnosis was found to be a significant predictor of daytime dysfunction, reinforcing the suggestion that mental health conditions (such as anxiety and depression) may contribute to poorer sleep quality outcomes in the autistic adult population.

Whether participants habitually napped, or not, significantly predicted sleep disturbances, daytime dysfunction, and daytime sleepiness. Therefore, individuals who reported habitually napping had higher sleep disturbances, higher daytime dysfunction, and increased daytime sleepiness as compared to those who reported not habitually napping. This finding is supported by evidence that habitual napping is associated with excessive daytime sleepiness in the general population [39]. Nonetheless, this finding is important as habitual napping may be perceived as an effective coping mechanism to alleviate daytime sleepiness in this population, when it could possibly be detrimental to overall sleep quality. Taken together, these findings tentatively suggest that habitual napping may have a negative impact on sleep quality through the potential reduction in nocturnal sleep pressure in an autistic adult population. However, this relationship may be bidirectional, with excessive daytime sleepiness resulting in napping or vice versa. That said, the finding that daytime sleepiness was not significantly associated with napping duration supports the former interpretation.

An association between age and sleep quality, with increased age being associated with decreased sleep latency and decreased sleep duration, was also observed. These findings partially support research in the general population that ageing is associated with reduced sleep quality [9,11,12]. However, they contrast with the finding that ageing is associated with increased sleep latency in the general population [10]. Of note, no association between age and the other sleep quality variables was observed. Given that poor sleep quality is a lifelong issue for autistic adults [55], one possibility is that their sleep quality never changes, which is why we do not see the same influence of age on sleep quality as in the general population.

Furthermore, the lack of any significant gender influence on sleep quality is at odds with findings in the general population, where females report more sleep problems than males and have a higher risk of developing insomnia [6,16]. Previous work has demonstrated that autistic adults experience a range of barriers with regards to seeking help for their sleep problems (e.g., not considering their sleep a health priority, reluctant to visit a HCP), regardless of the finding that females report higher sleep problems than males [56]. Consequently, it may be the case that these barriers outweigh any gender effects on sleep quality. In addition, emerging evidence suggests that, compared to the general population, more autistic adults identify as non-binary [57,58]. In the present study, participants were asked to report their current identified gender, as opposed to gender assignment at birth. Consequently, this may be one reason why gender differences were not found in this study compared to those found in the general population.

In addition, there were no significant associations between SES ranking and sleep quality. This finding is at odds with studies in the general population that found poorer sleep and a higher prevalence of sleep disturbances are more likely in individuals who report higher levels of social deprivation such as an unemployment and lower level of education [6,17,18,19,20,21,22]. Moreover, these findings do not support the reported associated between sleep quality and unemployment in autistic adults [41]. Extending the previous research, an association between sleep quality and SES ranking, not just one facet of SES (employment), was observed in the current study. This may be one reason why SES ranking was not found to significantly predict sleep quality in this study. Moreover, as mentioned above, comorbid conditions, such as anxiety, may override any effects of SES on sleep quality.

Regarding the influence of other lifestyle factors, the findings are in line with the suggestion that autistic adults preferences differ in terms of exercise activities and socialisation during exercise, and therefore factors like exercise may not reap the same benefits as in the general population [42].

### Limitations

Despite significant predictors emerging in the majority of our regression models, overall, these models explained a small proportion of the variance across PSQI subscales, global score, and daytime sleepiness (4.2–14.3%). In addition, all computed effect sizes indicated small-to-medium effects. It should also be noted that the Cronbach’s alpha value for the total PSQI was adequate at 0.068. Taken together, further research is needed to identify other factors that might diminish or improve sleep quality in this population. For example, gender, exercise, and alcohol, although found to influence sleep quality in the general population, did not influence sleep quality in our sample. To identify other important factors, more research is warranted from the perspective of autistic adults (e.g., focus groups) regarding what factors they believe contribute to their sleep quality.

Moreover, our sample was predominantly white (89.2%). Given previous work demonstrating poorer sleep quality in African-American and Latino individuals compared to White individuals [59], our findings may not accurately represent the experience of all autistic adults. Future work obtaining additional demographic variables, with a larger, more diversified sample is needed to investigate the contribution of other demographic and lifestyle factors on sleep quality in autistic adults.

Another limitation is that our findings may be attributed to the high percentage of our total sample who scored >5 on the PSQI (89.66%); demographic and lifestyle factors that influence participants with a sleep disorder/disturbance are likely going to differ from those who do not have these chronic sleep problems. This is supported by the finding that insomnia was a significant predictor of overall sleep quality. Moreover, the results suggested that habitual napping is a possible barrier to achieving good sleep quality in this population. In our total sample 21.91% of participants scored greater than >10 on the ESS, suggesting excessive daytime sleepiness. In the general population, napping is only beneficial when excessive daytime sleepiness is present. Future research should screen for sleep disorders in this population in order to decipher those who have chronic sleep problems from those who do not.

In addition, our findings were not compared to a control group (e.g., the inclusion of a neurotypical control group). This would have enabled us to better support our hypotheses that demographic and lifestyle predictors have a specific influence on sleep quality in autistic adults. Future research should consider recruitment of such groups for comparisons and to further develop this area of study.

It should also be acknowledged that information about self-diagnosis of other physical and mental conditions was self-reported (see Supplementary Materials Table S2). Consequently, there is no way to verify diagnoses in these participants. However, it has been demonstrated that individuals are able to provide reliable information on their own health and how it changes over time [60,61]. In addition, self-diagnosis was not used as a basis to include any mental or physical condition covariates in the analysis.

A final limitation relates to the ability to verify diagnosis via an online survey. To address this, participants were asked to provide information on their diagnosis (e.g., age of diagnosis, professional who gave the diagnosis, and month and year of the diagnosis) in order to potentially limit this issue. Further, participants who self-reported their autism diagnosis were also included in the sample. That said, McDonald [44] compared autistic adults self-diagnosed to autistic adults diagnosed by a HCP and found the inclusion of self-reported autistic adults in online surveys likely represents the “lost generation” of autistic adults, such as older females. In the present sample, there were twice as many females than males, aged between 18 and 73 years old. This demographic is likely a reflection of the recruitment strategy and collaboration with UK charitable organisations. Therefore, the sample included and represented both autistic adults with an HCP diagnosis and those who identified as self-diagnosed.

## 5. Conclusions

This study provides novel insight into what factors predict different facets of sleep quality and daytime sleepiness in an autistic adult population. Furthermore, this study highlights the similarities and differences with regards to factors that have been shown to predict sleep quality in the general population. In the present sample, it was found that insomnia and anxiety diagnosis predicted poorer sleep quality above and beyond demographic and lifestyle factors. In addition, partially similar results to general population findings for age only, and divergent results for habitual napping, were also observed. Although further research is needed to understand which factors predict sleep quality in this population, this study serves as a good starting point for targeting specific factors when developing tailored sleep treatments for this population. For example, the present study acknowledges differences in sleep issues in this population (e.g., sleep latency and daytime dysfunction). Moreover, the study highlights that addressing some factors may not work, as assumed, in the autistic adult population (e.g., napping) is warranted. This is pertinent for uncovering the specific needs and preferences of autistic adults and how sleep interventions should be tailored to this.

## Figures and Tables

**Table 1:** Summary of autistic adults’ demographic information.

Demographic Variables	*n*	*%*
Age		
18–29	164	33.3
30–39	140	28.4
40–49	124	25.2
50–59	53	10.8
60–69	10	2.0
70–79	1	0.2
Unknown/not reported	1	0.2
SES ranking		
1–2—Least deprived	14	2.8
3–4	51	10.3
5–6	85	17.2
7–8	117	23.7
9–10—Most deprived	152	30.8
Unknown/not reported	74	15.0
Education level		
Primary school	8	1.6
Secondary school up to 16 years	44	8.9
Higher, secondary or further education	120	24.3
Undergraduate degree	168	34.1
Postgraduate degree	142	28.8
Unknown/not reported	11	2.2
Ethnicity		
White	440	89.2
Black African	2	0.4
Black Caribbean	3	0.6
Mediterranean	1	0.2
Ashkenazi Jew	3	0.6
Indigenous	1	0.2
Hispanic	3	0.6
Romani	2	0.4
Latina	1	0.2
Asian	1	0.2
Mixed ethnicity	22	4.5
Unknown/not reported	14	2.8
Employment status		
Employed (full time)	130	26.4
Employed (part time)	70	14.2
Self-employed	53	10.8
Student	82	16.6
Retired	6	1.2
Unemployed looking for work	35	7.1
Unemployed not looking for work/unable to work	47	9.5
On disability allowance	56	11.4
Home maker	11	2.2
Unknown/not reported	3	0.6
Household income		
Less than £20,000	182	36.9
£20,000–£29,999	69	14.0
£30,000–£39,999	44	8.9
£40,000–£59,999	53	10.8
£60,000–£79,000	42	8.5
£80,000–£99,999	17	3.4
£100,000–£149,999	14	2.8
More than £150,000	8	1.6
Unknown/not reported	64	13.0

**Table 2:** Means and standard deviations of outcome measures and final sample size for each outcome measure.

Outcome Measures	Subscales	*M*	*SD*
PSQI	Subjective sleep quality (*n* = 493)	1.78	0.71
	Sleep latency (*n* = 480)	2.18	1.03
	Sleep duration (*n* = 483)	1.08	1.10
	Sleep efficiency (*n* = 472)	1.41	1.21
	Sleep disturbance (*n* = 493)	1.63	0.61
	Sleep medication (*n* = 493)	0.83	1.20
	Daytime dysfunction (*n* = 493)	1.79	0.77
PSQI total (*n* = 467)		10.67	3.99
ESS total (*n* = 493)		6.88	4.82

**Table 3:** Reported grouped median and interquartile range on significant sleep quality measures.

**Gender: Identifying as Male**				
	Male		Other (female and non-binary)	
Measures	Mdn	IQR	Mdn	IQR
PSQI Sleep Disturbance	1.49	1.00	1.66	1.00
PSQI Sleep Medication	0.33	1.00	0.57	2.00
**Gender: Identifying as Female**				
	Female		Other (male and non-binary)	
Measures	Mdn	IQR	Mdn	IQR
PSQI Sleep Duration	0.99	2.00	0.75	2.00
PSQI Sleep Medication	0.57	2.00	0.42	1.00
**Gender: Identifying as Non-Binary**				
	Nonbinary		Other (male and female)	
Measures	Mdn	IQR	Mdn	IQR
PSQI Sleep Duration	0.67	2.00	0.93	2.00
**Autism Diagnosis**				
	Diagnosed as autistic by an HCP		Self-diagnosed	
Measures	Mdn	IQR	Mdn	IQR
PSQI Subjective Sleep Quality	1.79	1.00	1.62	1.00
**Exercise Presence**				
	Exercise		No exercise	
Measures	Mdn	IQR	Mdn	IQR
PSQI Sleep Latency	2.30	2.00	2.48	1.00
PSQI Daytime Dysfunction	1.69	1.00	1.86	1.00
**Exercise Frequency**				
	Exercise frequently (≥3 a week)		Exercise infrequently (<3 a week)	
Measures	Mdn	IQR	Mdn	IQR
ESS Total Score	5.75	7.00	6.87	5.00
**Habitual Napping**				
	Habitual napper		Not a habitual napper	
Measures	Mdn	IQR	Mdn	IQR
PSQI Subjective Sleep Quality	1.89	1.00	1.72	1.00
PSQI Sleep Duration	1.24	2.00	0.81	2.00
PSQI Sleep Disturbance	1.87	1.00	1.54	1.00
PSQI Daytime Dysfunction	2.18	1.00	1.65	1.00
PSQI Total Score	11.73	5.00	10.02	6.00
ESS Total Score	9.65	8.00	5.27	7.00

Higher scores indicate poorer sleep quality. HCP = healthcare professional.

**Table 4:** Overview of significant associations between demographic and lifestyle variables and sleep quality measures.

Measures	Significant Associations with Measures	r_s_	U	*p*
PSQI Subjective Sleep Quality	Autism diagnosis	-	15,180.00	0.048
	Napping presence	-	19,896.50	0.028
PSQI Sleep Latency	Age	−0.09	-	0.043
	SES ranking	0.20	-	<0.001
	Exercise presence	-	25,341.00	0.032
PSQI Sleep Duration	Age	0.19	-	<0.001
	Gender: identifying as female	-	25,334.00	0.024
	Gender: identifying as nonbinary	-	13,998.00	0.036
	Napping presence	-	17,950.50	0.006
PSQI Sleep Disturbance	Age	0.12	-	0.009
	SES ranking	0.13	-	0.006
	Gender: identifying as male	-	19,216.00	0.009
	Napping presence	-	16,616.00	<0.001
PSQI Sleep Medication	Gender: identifying as male	-	19,229.00	0.007
	Gender: identifying as female	-	27,119.50	0.042
PSQI Daytime Dysfunction	SES ranking	0.16	-	0.001
	Exercise presence	-	26,606.50	0.028
	Napping presence	-	15,692.00	<0.001
PSQI Total Score	SES ranking	0.12	-	0.019
	Napping presence	-	16,334.00	0.003
ESS total	Exercise frequency	-	7385.50	0.047
	Napping presence	-	11,438.50	<0.001

**Table 5:** Significant predictors and coefficients from regression models.

Measures	Predictors	B	SE	β	*p*
PSQI Subjective Sleep Quality	Insomnia diagnosis	0.38	0.12	0.19	0.001
	Anxiety diagnosis	0.27	0.08	0.19	0.002
PSQI Sleep Latency	Anxiety diagnosis	0.33	0.13	0.16	0.010
	Insomnia diagnosis	0.51	0.18	0.17	0.004
	Age	−0.01	0.01	−0.14	0.019
PSQI Sleep Duration	Anxiety diagnosis	0.35	0.13	0.16	0.009
	Age	0.02	0.01	0.15	0.015
PSQI Sleep Efficiency	Anxiety diagnosis	0.36	0.15	0.15	0.016
	Insomnia diagnosis	0.42	0.21	0.12	0.047
PSQI Sleep Disturbance	Insomnia diagnosis	0.31	0.10	0.18	0.003
	Napping presence	0.23	0.09	0.16	0.007
	Anxiety diagnosis	0.15	0.07	0.12	0.042
PSQI Sleep Medication	Insomnia diagnosis	1.01	0.20	0.30	<0.001
PSQI Daytime Dysfunction	Depression diagnosis	0.24	0.11	0.15	0.026
	Napping presence	0.36	0.11	0.19	0.001
	Anxiety diagnosis	0.25	0.11	0.16	0.022
PSQI Total Score	Insomnia diagnosis	3.21	0.67	0.28	<0.001
	Anxiety diagnosis	1.71	0.48	0.21	<0.001
ESS total	Napping presence	4.18	0.64	0.37	<0.001

## Data Availability

The data that support the findings of this study are available on request from the corresponding author D.D. The data are not publicly available due to privacy reasons.

## Supplementary Materials

The following are available online at https://www.mdpi.com/article/10.3390/ijerph18189883/s1, Table S1: Summary of autistic adults’ demographic lifestyle characteristics, Table S2: Summary of autistic adults’ diagnoses, Table S3. Summary of autistic adults’ medication use. Table S4: Final sample size for each linear regression model.
