# Supplementary material for: Anxiety, Insomnia, and Napping Predict Poorer Sleep Quality in an Autistic Adult Population
Source: Int J Environ Res Public Health. 2021 Sep 19;18(18):9883. doi: 10.3390/ijerph18189883 (PMC8469045; doi:10.3390/ijerph18189883)
Supplement: Supplementary file 1 [file ijerph-18-09883-s001.zip › ijerph-1360473-supplementary.pdf]

Supplementary material

Table S1. Summary of autistic adults' demographic lifestyle characteristics

**Additional demographic variables**	***n***	**%**
Daily smoking habits		
Non-smoker	448	90.9
Less than one cigarette a day	6	1.2
1-9 cigarettes a day	16	3.2
10-19 cigarettes a day	17	3.4
20-39 cigarettes a day	6	1.2
Daily alcohol habits		
Non-drinker	212	43.0
Drink less than 1 unit per day	215	43.6
Drink between 1-2 units per day	40	8.1
Drink between 3-6 units per day	21	4.3
Drink between 7-9 units per day	4	0.8
Drink more than 9 units per day	1	0.2
Weekly exercise habits		
Everyday	47	9.5
At least 5 times a week	53	10.8
At least 3 times a week	88	17.8
At least once a week	78	15.8
At least once a month	12	2.4
Less than once a month	2	0.4
No exercise	213	43.2
Number of naps per day		
Non-napper	371	75.3
1-2 naps	119	24.1
3-4 naps	3	0.6
Duration of naps		
Less than 15 minutes	9	7.4
15-30 minutes	24	19.7
30-59 minutes	35	28.7
60-89 minutes	22	18.0
90-119 minutes	18	14.8
More than 119 minutes	14	11.5

Duration of naps included the n = 122 who regularly nap during the day.

Table S2. Summary of autistic adults' diagnoses.

**Diagnoses**	**Diagnosed by an HCP**		**Self-diagnosed**		**Total**
	n	**%**	***n***	**%**	**%**
Mental health diagnoses					
**Depression**	**261**	**52.9**	60	12.2	65.1
**Anxiety**	**260**	**52.7**	80	16.2	69.0
ADHD	85	17.2	106	21.5	38.7
PTSD	82	16.6	112	22.7	39.4
OCD	35	7.1	53	10.8	17.8
Bipolar disorder	15	3.0	15	3.0	6.1
Personality disorder	15	3.0	1	0.2	3.2
Eating disorder	10	2.0	3	0.6	2.6

Brain injury	7	1.4	11	2.2	3.7
Dissociative disorder	5	1.0	3	0.6	1.6
Substance use disorder	5	1.0	17	3.4	4.5
Schizophrenic spectrum	4	0.8	5	1.0	1.8
Tourette's syndrome	3	0.6	13	2.6	3.2
Psychosis	2	0.4	-	-	0.4
Specific phobia	2	0.4	-	-	0.4
Excoriation disorder	1	0.2	1	0.2	0.4
Panic disorder	1	0.2	-	-	0.2
Pathological demand avoidance	1	0.2	1	0.2	0.4
Pre-menstrual dysphoric disorder	1	0.2	1	0.2	0.4
Alexithymia	-	-	2	0.4	0.4
Body dysmorphic disorder	-	-	1	0.2	0.2
Cyclothymia	-	-	1	0.2	0.2
Dementia	-	-	3	0.6	0.6
Physical health diagnoses					
Gastrointestinal	113	22.9	53	10.8	33.7
Respiratory	107	21.7	10	2.0	23.7
Head, eyes, ears, nose and throat	96	19.5	35	7.1	26.6
Musculoskeletal	93	18.9	29	5.9	24.7
Dermatological	85	17.2	25	5.1	22.3
Mobility	85	17.2	34	6.9	24.1
Neurological	58	11.8	17	3.4	15.2
Cardiovascular	32	6.5	11	2.2	8.7
Endocrine/metabolic	32	6.5	5	1.0	7.5
Chronic pain/fatigue	27	5.5	6	1.2	6.7
Autoimmune	15	3.0	9	1.8	4.9
Gynaecological	12	2.4	1	0.2	2.6
Allergy	11	2.2	-	-	2.2
Genitourinary	8	1.6	28	5.7	7.3
Blood/lymphatic	2	0.4	-	-	0.4
Cancer	2	0.4	-	-	0.4
Congenital	2	0.4	-	-	0.4
Obesity	1	0.2	-	-	0.2
Processing diagnoses					
Sensory processing disorder	75	15.2	177	35.9	51.1
Speech and language disorders	29	5.9	33	6.7	12.6
Auditory processing disorder	4	0.8	9	1.8	2.6
Visual and perceptual disorders	4	0.8	2	0.4	1.2
Learning difficulties/genetic diagnoses					
Dyspraxia	38	7.7	35	7.1	14.8
Dyslexia	35	7.1	29	5.9	13.0
Dyscalculia	21	4.3	40	8.1	12.4
Social communication disorder	20	4.1	25	5.1	9.1
Genetic	8	1.6	5	1.0	2.6
Dysgraphia	7	1.4	21	4.3	5.7
Global developmental delay	7	1.4	7	1.4	2.8
Sleep diagnoses					
**Insomnia**	**66**	**13.4**	220	44.6	58.0
Obstructive Sleep Apnoea	28	**5.7**	53	10.8	16.4
Restless Leg Syndrome	24	4.9	130	26.4	31.2

Parasomnia	20	4.1	87	17.6	21.7
Circadian Rhythm Disorder	13	2.6	123	24.9	27.6
Somnambulism	7	1.4	28	5.7	7.1
Sleep disordered breathing	6	1.2	51	10.3	11.6
Periodic Limb Movement Disorder	4	0.8	50	10.1	11.0
Hypersomnia	2	0.4	-	-	0.4
REM Behaviour disorder	2	0.4	55	11.2	11.6
Narcolepsy	1	0.2	18	3.7	3.9

HCP = healthcare professional, Genetic conditions (e.g., Down's syndrome, Fragile X Syndrome). % represent the number of participants with one or more diagnoses in the category (e.g., one or more cardiovascular diagnoses).

Table S3. Summary of autistic adults' medication use.

**Medication use**	*n*	%
Prescribed medication		
Antidepressants	164	33.3
Allergy	72	14.6
Anti-anxiety	66	13.4
Cardiovascular	63	12.8
Sleep	62	12.6
Endocrine/metabolic	38	7.7
Stimulants/ADHD	36	7.3
Respiratory	32	6.5
Neurological	31	6.3
Chronic pain/fatigue	29	5.9
Gastrointestinal	22	4.5
Anti-psychotics	19	3.9
Mood stabilisers	17	3.4
Autoimmune	6	1.2
Blood/lymphatic	5	1.0
Musculoskeletal	4	0.8
Dermatologic	3	0.6
Head, ears, eyes, nose and throat	2	0.4
Cancer	1	0.2
Self-medication		
Cannabis/CBD oil	24	4.9
Vitamin/mineral supplements	11	2.2
OTC painkillers	8	1.6
Amino acid supplements	7	1.4
Herbal supplements	7	1.4
Nutritional/dietary supplements	6	1.2
Alcohol	3	0.6
Caffeine	3	0.6
OTC anti-sickness	2	0.4
OTC antihistamines	1	0.2
Melatonin use		
No, never	320	64.9
No, but have previously	116	23.5
Yes, currently	57	11.6

[1] % represent the number of participants taking one or more medications in the category (e.g., taking one or more cardiovascular medications).

Table S4. Final sample size for each linear regression model

**Outcome measures**	***Subscales***	***N***	**%**
PSQI	Subjective sleep quality	279	56.6
	Sleep latency	271	55.0
	Sleep duration	275	55.8
	Sleep efficiency	267	54.2
	Sleep disturbance	279	56.6
	Sleep medication	279	56.6
	Daytime dysfunction	279	56.6
PSQI total		265	53.8
ESS total		279	56.6
